# Implementation and outcomes of a comprehensive emergency care curriculum at a low-resource referral hospital in Liberia: A novel approach to application of the WHO Basic Emergency Care toolkit

**DOI:** 10.1371/journal.pone.0282690

**Published:** 2023-03-15

**Authors:** Lane Epps, Anu Ramachandran, Sojung Yi, Alexander Mayah, Taylor Burkholder, Michael Jaung, Ahson Haider, Paul Wesseh, John Shakpeh, Corey Bills, Kayla Enriquez

**Affiliations:** 1 Department of Emergency Medicine, University of California San Francisco, San Francisco, CA, United States of America; 2 Department of Emergency Medicine, University of Southern California, Los Angeles, CA, United States of America; 3 Department of Emergency Medicine, Baylor College of Medicine, Houston, TX, United States of America; 4 San Francisco State University, San Francisco, CA, United States of America; 5 Redemption Hospital, Monrovia, Liberia; 6 Department of Emergency Medicine, University of Colorado School of Medicine, Aurora, CO, United States of America; University of Sharjah, UNITED ARAB EMIRATES

## Abstract

**Background:**

Emergency care is vital in low- and middle-income countries (LMICs) but many frontline healthcare workers in low-resource settings have no formal training in emergency care. To address this gap, the World Health Organization (WHO) developed *Basic Emergency Care (BEC)*: *Approach to the acutely ill and injured*, a multi-day, open-source course for healthcare workers in low-resource settings. Building on the *BEC* foundation, this study uses an implementation science (IS) lens to develop, implement, and evaluate a comprehensive emergency care curriculum in a single emergency facility in Liberia.

**Methods:**

A six-month emergency care curriculum consisting of *BEC* content, standardized WHO clinical documentation forms, African Federation of Emergency Medicine (AFEM) didactics, and clinical mentorship by visiting emergency medicine (EM) faculty was designed and implemented using IS frameworks at Redemption Hospital, a low-resource public referral hospital in Monrovia, the capital of Liberia. Healthcare worker performance on validated knowledge-based exams during pre- and post-intervention testing, post-course surveys, and patient outcomes were used to evaluate the program.

**Results:**

Nine visiting EM physicians provided 1400 hours of clinical mentorship and 560 hours of didactic training to fifty-six Redemption Hospital staff over six-months. Median test scores improved 20.0% (p<0.001) among the forty-three healthcare workers who took both the pre- and post-intervention tests. Participants reported increased confidence in caring for medical and trauma patients and comfort performing emergency care tasks on post-course surveys. Emergency unit (EU)/Isolation unit (IU) mortality decreased during the six-month implementation period, albeit non-significantly. Course satisfaction was high across multiple domains.

**Discussion:**

This study builds on prior research supporting WHO efforts to improve emergency care globally. *BEC* implementation over a six-month timeframe using IS principles is an effective alternative strategy for facilities in resource-constrained environments wishing to strengthen emergency care delivery.

## Introduction

Emergency care is a vital part of effective healthcare systems. In low- and middle-income countries (LMICs) identification and treatment of life-threatening conditions are critical because of the high burden of time-sensitive injuries and illnesses [1]. *Disease Control Priorities 3rd edition (DCP3)* estimates that emergency care can address the conditions that account for approximately half of deaths in LMICs such as diarrheal diseases, lower respiratory infections, and trauma [2], but frontline healthcare workers in these settings often lack formal training in emergency care [1,3,4]. Emergency care courses developed for low-resource settings often rely on partnerships with high-income countries, are limited in scope (e.g. specifically focusing on trauma, resuscitation, ultrasound, or toxicology), and have targeted audiences (e.g. intended for EMS personnel, nurses, or surgical residents) [5–12]. Other widely used programs, Advanced Trauma Life Support (ATLS) for example, are cost-prohibitive and require investment in training infrastructure and medical equipment that are not readily available in resource-constrained settings. Although the cost of standardized programs can be reduced by training local instructors, there are still significant barriers to the widespread implementation of such programs [13].

To address the need for context-specific and accessible emergency care education, the World Health Organization (WHO), International Committee of the Red Cross (ICRC), and International Federation of Emergency Medicine (IFEM) developed *Basic Emergency Care (BEC)*: *Approach to the acutely ill and injured*, an open-source emergency care training program for frontline healthcare workers in low-resource settings [14]. The five-day course consists of didactic training complemented by a workbook, clinical skill-building sessions, and practical scenarios. Previous pilot programs of the course in Uganda, Tanzania, and Zambia were well received, improved participant knowledge-based test performance, and increased healthcare worker confidence performing key emergency care skills [15]. Effects on patient outcomes or quality indicators have not yet been assessed. *BEC* is an important resource for frontline healthcare workers in low-resource settings, but may need to be augmented for individuals with limited prior emergency care training or experience. As such, there is a significant opportunity to develop more robust and comprehensive programs by building on the *BEC* foundation.

Liberia is a low-income nation in West Africa [16] that has faced significant challenges developing effective emergency care systems due to prolonged civil conflict, the Ebola epidemic, and slow economic recovery. During initial rebuilding efforts following the Liberian Civil Wars (1989–2003), the Liberian Ministry of Health and Social Welfare (MOHSW) identified emergency care as an essential health service in the Basic Package of Health Services (BPHS) [17]. BPHS stipulated that all healthcare facilities should be able to initially stabilize and manage patients with hemorrhage, shock, respiratory distress, anaphylaxis, and seizures. The 2011 Essential Package of Health Services (EPHS), defined a tiered model of emergency care capabilities for community health clinics, county hospitals, and tertiary healthcare centers [18]. Despite this regulation, healthcare facilities had challenges delivering adequate emergency services, with studies estimating that only a quarter of people had access to emergency care in some rural counties [19]. In 2014, the West African Ebola epidemic forced many operational facilities to close to mitigate the spread of Ebola Virus Disease (EVD) [20]. As a result, emergency services steeply declined during this time period [21]. Between 2015–2016, the MOHSW, in partnership with the WHO, implemented the Safe and Quality Services package (SQS) to address identified gaps in triage, emergency care, and infection prevention and control (IPC) during the Ebola epidemic [22]. As part of the program, approximately 15,000 healthcare workers in all fifteen counties in Liberia were given basic emergency care training. Although SQS improved healthcare worker confidence in emergency management and surveillance, widespread inadequacies in the overall state of emergency care in Liberia were identified. Additionally, as non-governmental organizations (NGOs) and other aid organizations phased out in the post-Ebola recovery period, healthcare worker emergency care knowledge retention decreased without a plan to implement SQS as a sustainable program.

Given these unique historical gaps in emergency care training in Liberia, an implementation science (IS) framework was used to develop, implement, and evaluate a comprehensive emergency care program based on open-source WHO and African Federation of Emergency Medicine (AFEM) curricula in order to support frontline healthcare workers in a single emergency facility in Liberia. Here, we describe the development, implementation strategy, and preliminary outcomes of the program. We demonstrate that *BEC* implementation over a six-month timeframe as part of a robust program is an effective alternative strategy for facilities in resource-constrained environments wishing to strengthen emergency care delivery.

## Methods

### Setting

Redemption Hospital is a 175-bed secondary level referral center and the only public hospital serving Monrovia, the capital of Liberia. It has a 10-bed emergency unit (EU), a small isolation unit (IU), maternal and pediatric wards, a general inpatient medical ward, and outpatient services. In June 2014, Redemption Hospital was closed to prevent EVD transmission after twenty staff were infected, twelve of whom died from the disease [23]. The hospital reopened several months later after extensive decontamination, renovation, and development of IPC practices such as universal temperature checks, EVD screening, and proper waste management. Despite improvements in the post-Ebola era, critical gaps in infrastructure, medical equipment, training, and human-resources severely limit the quality of emergency care provided. Food and medication are often supplied by patients’ family members, there is unreliable access to clean water, and imaging and laboratory capabilities are minimal. At the time of program development, the EU and IU were staffed by one general practitioner as well as several physician assistants, nurses, and nursing assistants, all of whom had limited training in emergency care. Documentation was highly variable with no standardized format or content.

### Implementation science process

**Fig 1:**
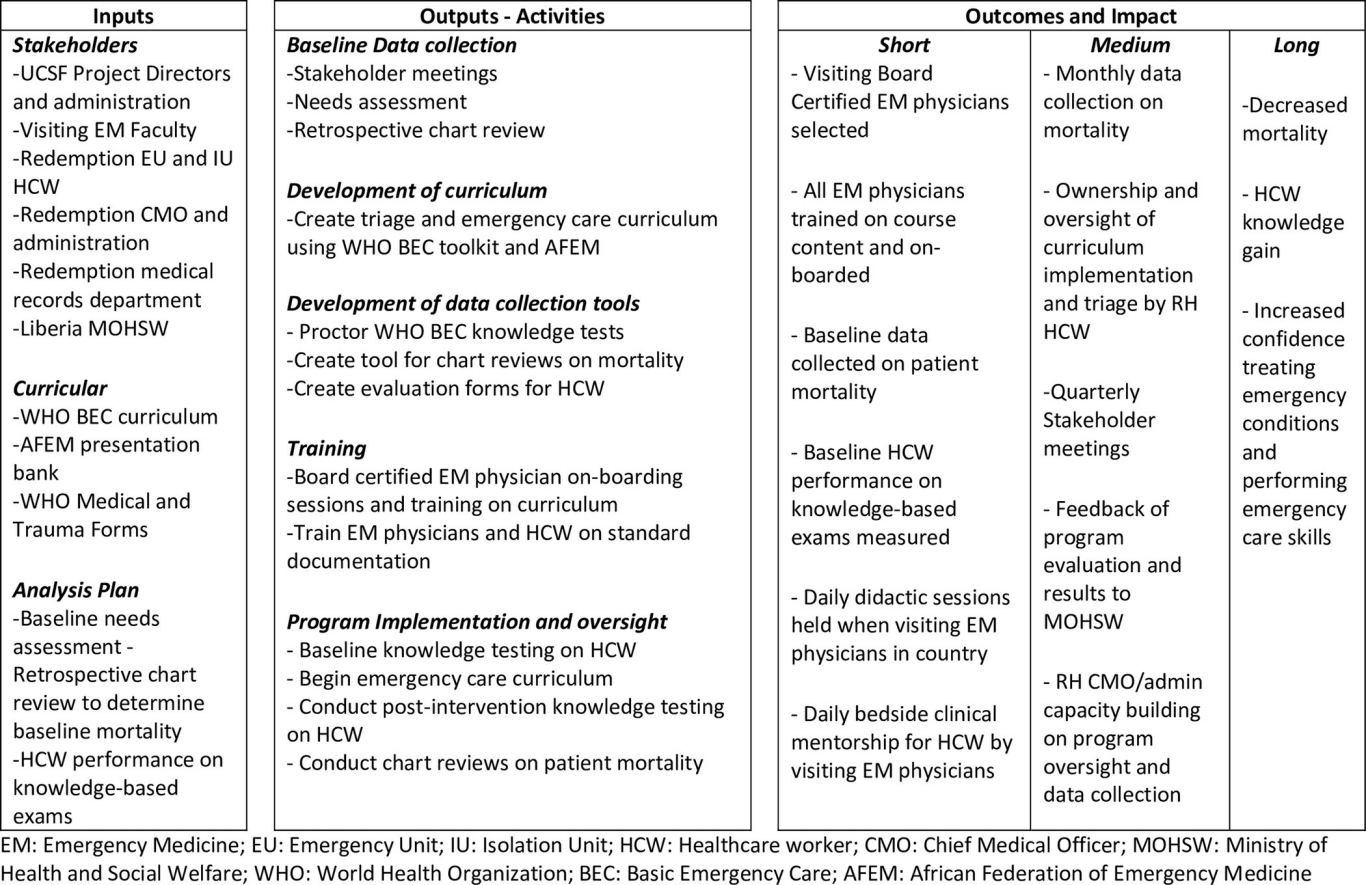
Implementation research logic model.

To understand why gaps in emergency care knowledge and practice existed after the implementation of SQS, the flexible, non-linear approach of IS was used to design and implement a comprehensive six-month emergency care program. IS strategies, including an Implementation Research Logic Model (IRLM) [24] and the *Reach*, *Effectiveness*, *Adoption*, *Implementation*, *and Maintenance* framework (RE-AIM) [25,26] were used to facilitate translation of evidence-based practices into clinical practice. An IRLM was created to depict shared relationships among resources, activities, outputs, outcomes, and impacts of the program and promote common understanding to all stakeholders (Fig 1). The model also identified programmatic responsibilities and ensured a method for accountability throughout the intervention. Given the comprehensive emergency care program was reliant on behavior change from frontline healthcare workers, the RE-AIM framework was used to evaluate programmatic interventions. By evaluating indicators in the various socio-ecological dimensions of the framework, the impact of the educational intervention could be better understood and further enhanced.

### Pre-implementation planning and program development

The emergency care program was created over a one-year span in 2018. Initial planning of the intervention focused on engaging key stakeholders and identifying barriers to healthcare worker behavior change. The WHO in-country lead, Minister of Health, County Chief Medical Officer, Chief Medical Officer at Redemption Hospital, Emergency Unit Nursing Director, and hospital administration were all involved in the programmatic planning and implementation. Feedback from frontline healthcare workers was also solicited during discussions of the intended impacts of the program on clinical documentation, patient care, and patient mortality. All key stakeholders took part in developing the program and helped ensure it met healthcare worker needs, was appropriate for implementation, and did not interfere with patient care. Initial meetings addressed gaps in education based on both anecdotal information and data from previous program implementation. Taking this information into account, the program implemented was designed to address these gaps and meet the local needs of the hospital and frontline workers. Key stakeholders were involved in each step of programming from deciding how to implement the curriculum, to specific curriculum supplemented and taught via AFEM open source lectures. Regular updates were relayed to all stakeholders and final data was shared upon completion of the program. All healthcare workers consented for voluntary participation in the program.

### Implementation strategy

WHO *BEC* content and lectures from the African Federation of Emergency Medicine (AFEM) open-source lecture bank [27] were selected based on clinical relevance for specific communicable and non-communicable disease pathologies commonly seen in Liberia and compiled for didactic programming. AFEM lectures were edited by clinicians with prior work experience in Liberia to ensure appropriate teaching on available resources and treatments. The intensive, 5-day *BEC* implementation period was adapted based on the clinical environment at Redemption Hospital. Given that there were few healthcare workers, short, on shift clinical didactic sessions were held two to three times a day to minimize disruptions to patient care. *BEC* was supplemented with AFEM content based on stakeholder requests for further educational material on common disease entities in Liberia. To facilitate shorter didactic sessions and additional AFEM content, the curated didactic curriculum for Redemption Hospital spanned over six-months broken into monthly blocks based on organ system and common chief complaints seen in Liberia. Triage had a dedicated curriculum integrated into the monthly content to ensure continual reinforcement and practice.

The WHO Standardized Clinical Forms for Medical and Trauma Patients [28] and Redemption Hospital Triage Form, which was adapted from existing WHO tools (S1 Appendix), were taught to all healthcare workers in the EU and IU over the first month of programming to ensure complete understanding of form utilization. All forms were then used for documentation throughout the six-month duration of the program. An additional one-year supply of WHO clinical forms was also provided to Redemption Hospital to help maintain documentation practices following completion of the program.

At the time of program development, there were no long-standing board-certified EM physicians working in Liberia. Nine visiting board-certified EM faculty were selected to facilitate the program based on their experience working clinically in low-income countries and their comfort with *BEC* and AFEM curricula. Visiting EM faculty were oriented via a series of phone calls and written guidelines, had frequent check-ins with the principal investigators while in-country, and 24-hour access to an in-country research assistant. While on-site, visiting faculty worked between five and six, eight-hour shifts per week for two to four weeks in the EU and IU at Redemption Hospital, conducting daily didactic sessions and providing clinical mentorship. When problems or delays occurred in teaching schedules or clinical shifts, the curriculum was adjusted to accommodate for unforeseen events. Visiting faculty members functioned as teachers and mentors to healthcare workers and were not intended to primarily care for patients presenting to the EU. They delivered the curriculum and worked alongside providers helping them make clinical decisions, utilize critical thinking, practice implementing knowledge and skills, and deliver adequate care based on the curriculum being taught. Once visiting EM physicians returned from Liberia, feedback was solicited through exit interviews to improve programming.

### Program evaluation and data collection

The RE-AIM framework [25,26] was used to evaluate the six-month program because of its attention to identifying the educational intervention’s target population (frontline healthcare workers) and appropriate setting (EU and IU), with the goal of successful programmatic implementation resulting in healthcare worker emergency care knowledge acquisition, increased confidence in the management of medical and trauma patients, and improved comfort performing emergency care skills.

A previously validated WHO *BEC* knowledge-based exam was administered prior to program implementation to assess baseline emergency care knowledge. After completion of the program, a distinct post-course WHO *BEC* knowledge-based exam was proctored to assess emergency care knowledge acquisition. A survey was also administered to all participants to assess course satisfaction, confidence treating emergency conditions, comfort performing emergency care skills, and gather feedback for future programming (S2 Appendix). On completion of programming, all participants were invited to attend a wrap-up session in which preliminary data were presented.

At Redemption Hospital, initial patient registration is recorded in a ledger that summarizes basic demographic and clinical data. Retrospective review of the EU/IU ledger in the year prior to course implementation (January 2018—December 2018) and in the six months during course implementation (January 2019—June 2019) was performed to compare EU/IU mortality. Patient disposition was stored on a password-protected Excel sheet with encryption for analysis.

The study, including chart review, was reviewed and deemed exempt by the National Research Ethics Board of the Liberian Ministry of Health and Social Welfare and the Institutional Review Board of the University of California San Francisco. All participants gave oral consent to participate in the course. Patient consent was waived for the chart review portion of the study.

### Data analysis

Descriptive and exploratory data analyses, not presented here, were conducted prior to program implementation to determine the most common patient chief complaint and available medical equipment and infrastructure to drive overall program content. Descriptive data on program provider enrollment is presented as numbers and percentages. Provider self-assessments of knowledge and comfort with emergency care tasks, are reported as means with standard deviations and compared using paired t-tests. Scores on pre- and post-implementation validated exams are compared by Wilcoxon Rank Sum Tests, and stratified by provider type, location, and question category. Clinical outcomes are limited to EU/IU mortality, with additional clinical metrics, such as time to antibiotic administration, reported in a subsequent study. EU/IU mortality was calculated as the number of adult patients who died in the EU or IU, not including those who died before arrival, divided by the total number of adult patients seen in the EU or IU in the given study period as measured by EU/IU ledger records. EU/IU mortality was compared pre-implementation and cumulatively during implementation, via the chi-square (χ2) statistic. Course satisfaction surveys (collected as Likert-scale data) are presented as means and standard deviations. Statistical analyses were performed using STATA 17 [29].

## Results

A total of fifty-six frontline healthcare workers participated in the 6-month course. The majority of participating staff were nurses or physician assistants (N = 42, 75.0%); additional staff included hygienists and nursing assistants (N = 14, 25.0%). Emergency and acute care at the facility under study included both an emergency (EU) and isolation unit (IU); over two-thirds of providers worked primarily in the EU (N = 28, 67.9%) and 18 (32.1%) worked in the IU.

Pre-implementation self-assessed knowledge and comfort with performing tasks associated with the key domains of the course ranged on average from 3.62–4.13 (on a 5-point Likert scale) (Table 1). On average, providers were least sure of their knowledge and least comfortable performing tasks associated with the approach to the patient with altered mental status (3.85, SD: 0.66; 3.62, SD: 0.72, respectively) (Table 1). Post-implementation, providers reported statistically significant increased comfort in performing all associated tasks and felt more comfortable caring for both medical and trauma patients, as compared to pre-course.

**Table 1:** Pre- and post-course self-assessment*.

Characteristic	Pre-Implementation Mean (SD)	Post-Implementation Mean (SD)	P-value
Knowledge of: Triage Airway Assessment Breathing Circulation Mental Status Documentation	4.13 (0.57) 4.00 (0.82) 4.25 (0.50) 4.05 (0.62) 3.85 (0.66) 4.05 (0.62)	-- -- -- -- -- --	
Comfort performing tasks associated with: Triage Airway Breathing Circulation Mental Status Documentation	3.86 (0.82) 4.00 (0.84) 4.14 (0.72) 4.00 (0.67) 3.62 (0.72) 3.97 (0.88)	4.54 (0.51) 4.49 (0.51) 4.53 (0.51) 4.51 (0.51) 4.37 (0.54) 4.49 (0.51)	<0.001 0.002 0.012 0.003 <0.001 <0.001
Comfort taking care of critically ill: Medical patients Trauma patients	4.14 (0.81) 3.80 (0.83)	4.61 (0.49) 4.51 (0.51)	<0.001 <0.001
Practice changed in: Triage ABC Documentation Quality Mortality	-- -- -- -- --	4.55 (0.74) 4.49 (0.76) 4.54 (0.76) 4.64 (0.53) 4.57 (0.50)	
Overall impression of care given	3.73 (0.87)	4.34 (0.68)	<0.001

*All values measured via Likert scale from 1 (strongly disagree) to 5 (strongly agree).

Over the 6-month implementation period, nine visiting board-certified EM faculty presented 560 hours of didactic content to Redemption Hospital staff and provided 1,400 hours of bedside clinical teaching and mentorship. In total, sixty individual lectures were presented to EU staff and fifty-two to IU staff.

Overall, the median score on validated pre-course assessments was 48.0 (IQR: 36.0–52.0) among fifty-three individuals. The median score on a distinct validated post-course assessment was 68.0 (IQR: 48.0–80.0) among forty-six participants (Table 2). Significantly improved scores were noted between the forty-three participants that took both the pre- and post-course tests (p-value <0.001) (Table 2). While significant improvement was made among providers in both the EU and IU, median differences in pre- vs. post-course tests were significantly greater among IU staff (12% vs. 48%) (Table 2).

**Table 2:** Pre- and post-test course scores total, by location and provider type.

	Pre-test Median (IQR)	Post-test Median (IQR)	P-value
Total	48.0 (36.0–52.0)	68.0 (48.0–80.0)	<0.001
Location: Emergency Unit (EU) Isolation Unit (IU)	48.0 (36.0–52.0) 40.0 (32.0–48.0)	60.0 (48.0–68.0) 88.0 (80.0–88.0)	0.003 0.002
Provider Type: Physician Assistant/Nurse Other	48.0 (40.0–52.0 36.0 (28.0–48.0)	68.0 (54.0–84.0) 54.0 (48.0–68.0)	<0.001 0.016

Pre- and post-course test scores were also stratified by question type. There were significant improvements across all domains, with the largest median difference in pre- and post-course scores in questions about difficulty in breathing (Table 3).

**Table 3:** Pre- and post-course test scores total, by question type.

	ABCDE*	AMS*	DIB*	Shock*	Trauma*
Pre-Course Median (IQR)	33.3 (16.5–66.7)	40.0 (20.0–60.0)	33.3 (0–33.3)	50.0 (50.0–63.3)	50.0 (33.3–50.0)
Post-Course Median (IQR)	50.0 (50.0–100)	60.0 (60.0–100)	66.7 (66.7–100)	86.0 (57.0–100)	75.0 (66.7–83.3)
P-value	0.002	<0.001	<0.001	<0.001	<0.001

*N = 43; ABCDE: Airway, Breathing, Circulation, Disability, Exposure; AMS: Altered Mental Status; DIB: Difficulty in Breathing.

Overall course satisfaction was high across multiple domains (Table 4).

**Table 4:** Overall course satisfaction of participants*.

Question	Mean (SD)
I am satisfied with the content (the specific lecture topics) of this course	4.68 (0.47)
I am satisfied with the teaching methods (bedside teaching/didactic lectures) of this course	4.60 (0.50)
I am satisfied with the trainers/faculty members who were part of this course	4.56 (0.55)
I am satisfied with the organization of this course	4.58 (0.54)
I liked the format (continued faculty involvement, informally at bedside) of this course	4.49 (0.51)

*Satisfaction measured via Likert scale from 1 (strongly disagree) to 5 (strongly agree).

Those with a post-course test score greater than 60% were more likely to strongly agree that the quality of emergency care at Redemption Hospital improved (80.6% vs. 16.1%; p value = 0.006); and more likely to strongly hypothesize that EU/IU mortality improved (64.5% vs. 33.3%; p-value = 0.065), though this association was not significant. Participants also provided free-response comments to aid in future course development and EU/IU improvements. In general, comments suggested increased training and support for systems improvements.

Comprehensive evaluation of the program was conducted using the IS RE-AIM framework (Table 5). Overall, participants attended more than half of all lectures presented. The majority of participants demonstrated an improvement in knowledge-based test scores and self-reported rating of their clinical knowledge and skills in at least one domain. Not all participants answered the self-reported rating questions concerning clinical knowledge and skills.

**Table 5:** Evaluation of comprehensive emergency care program using the IS RE-AIM framework.

Domain	Quantitative findings	Qualitative (contributing factors)
**R**each	Total number of course participants = 56 Mean percent of total lectures attended = 54% Mean percent of *BEC* lectures attended = 68%	All EU and IU staff were invited to participate in the program Lectures were repeated throughout the course to accommodate varying schedules
**E**ffectiveness	Percent of participants with improvement in knowledge-based test scores = 81.4% (35/43)	Key content emphasized throughout lectures and during bedside clinical mentorship
	Percent of participants with post-course improvement in self-reported self-efficacy in at least one domain = 71.8% (28/39)	Bedside clinical mentorship incorporated to improve diagnostic and procedural skills relating to core emergency care domains
	Overall course satisfaction = 93.6% (4.68/5)	AFEM lectures adapted to highlight content specific to Liberia Multiple approaches to serve different learning needs EM faculty recruited with experience working in LMICs Adapted to participant feedback
	Practice changed in: Triage = 91.0% (4.55/5) ABC = 89.8% (4.49/5) Documentation = 90.8% (4.54/5)	Clinical mentorship to facilitate uptake of curriculum and standardized triage and documentation implemented as part of the program
**A**doption	Total number of visiting EM faulty = 9 Hours of Didactic Content = 560 Hours of clinical mentorship = 1400	Wide recruitment of dedicated visiting EM faculty In-country research assistant support Weekly meetings with PIs to evaluate course progress
**I**mplementation	100% of *BEC* content taught (divided into 11 lectures) 41 AFEM lectures taught with minor edits for clinical context in Liberia 2 *BEC* charting documentation tools implemented 1 triage tool implemented	Board certified EM physicians teaching didactics with bedside clinical mentorship for reinforcement, 3-week training on standard documentation
**M**aintenance	1 year of standardized charting documents supplied Knowledge retention not assessed	In-country research assistant conducting ad-hoc site check-ins and replenishment of standardized charting documents Limitation due to COVID-19 pandemic travel restrictions

IS: Implementation Science; RE-AIM: Reach, Effectiveness, Adoption, Implementation, Maintenance; EM: Emergency Medicine; EU: Emergency Unit; IU: Isolation Unit; AFEM: African Federation of Emergency Medicine; *BEC*: Basic Emergency Care.

Excluding patients who died prior to arrival, 1,958 and 1,523 patient encounters recorded in the EU/IU ledger were reviewed in the pre-implementation and during-implementation study periods respectively. EU/IU adult mortality did not change during course implementation (pre: 5.9% vs. during: 5.4%; p = 0.527).

## Discussion

The goal of this study was to develop, implement, and evaluate a comprehensive emergency care program to support frontline healthcare workers in a single public hospital in Liberia. Several IS concepts including an Implementation Science Logic Model (ISLR) and the Reach, Effectiveness, Adoption, Implementation, and Maintenance (RE-AIM) framework were used for planning and evaluation of the six-month program. The program incorporated open-source resources including WHO *BEC* content, AFEM lectures, standardized documentation with existing and modified WHO clinical forms, and clinical mentorship by visiting EM faculty. Overall, the program increased healthcare worker performance on validated knowledge assessments, confidence in treating critically ill medical and trauma patients, and comfort with performing all emergency care tasks. Emergency Unit (EU) and Isolation Unit (IU) mortality decreased during the course implementation period, although not significantly. This program offers an alternative implementation strategy for the WHO *BEC* toolkit that allows for curriculum adjuncts and further clinical support over a longer period. Implementing such a program under the frameworks of IS ensured continual assessment of factors that contributed to the translation of evidence-based practices into clinical practice.

The effectiveness of the program was assessed first by healthcare worker knowledge acquisition, as measured by differences in validated pre- and post-course exams. Improvement in performance on knowledge-based assessments was comparable to pilot programs of *BEC* in Zambia, Tanzania, and Uganda [15]. In these prior iterations, healthcare worker knowledge acquisition was assessed over a shorter time period of less than five days. Although the described programming in Liberia was not specifically designed to test knowledge retention, the results suggest that participants had longer-term recall of key concepts given that course content was taught over multiple months prior to administration of post-course exams. Healthcare workers at Redemption Hospital also had lower pre-course exam medians than pilot *BEC* participants, 48.0% as compared to 60.5%, likely due to the lower degree of existing emergency care training in Liberia. Physician assistants and nurses had higher pre-course test scores than participants with lower levels of training, but all providers had significant improvement on post-course exams, which suggests that the program was relevant for the wide variability in the level of training and experience at Redemption Hospital. When stratified by practice setting, IU staff performed better on post-course exams than did EU staff. Multiple factors may have contributed to this finding including better testing conditions in the IU, which often had fewer patients and disruptions than the EU, in addition to a more consistent learning environment over the course of the curriculum.

In addition to the pilot *BEC* programs in Zambia, Tanzania, and Uganda, additional iterations have been implemented in emergency facilities in Nigeria, Sierra Leone, Ukraine, and Kiribati and adapted for each clinical context [30–33]. In these cases, knowledge acquisition was similarly measured by comparing pre- and post-course assessments. In total, 161 healthcare workers participated in the additional BEC programming and had a mean score improvement of 15.8% (weighted average), again comparable to the findings in this study. In Tanzania specifically, additional educational supplements including clinical cases and a mobile phone app for easy reference have also been explored but did not improve performance on post-course assessments [34,35].

To our knowledge, this is the first program to incorporate *BEC* into a longer, comprehensive program delivered over a six-month period. Additionally, this is the first *BEC* implementation in Liberia, either as a standalone program or as part of a larger initiative. Besides the country-wide SQS program, only a few emergency care training courses in Liberia have been described. Basic life support (BLS), advanced cardiovascular life support (ACLS), pediatric advanced life support (PALS), and a modified neonatal resuscitation (NRP) curriculum were previously taught by American Heart Association healthcare providers during a two-day program at a rural hospital in Liberia [36]. Several emergency care modules were also developed by an academic medical consortium in partnership with a local non-governmental organization, Health Education and Relief Through Teaching (HEARTT). This specific program was part of an effort to deliver emergency care at John F. Kennedy Medical Center (JFKMC) in the post-conflict era that ended during the Ebola epidemic [37]. As many of these partnerships have dissolved and NGOs have left Liberia, frontline healthcare workers have needed to develop emergency care knowledge and skills to be able to work independently. This study is an example of how IS frameworks can be used to implement *BEC* as part of a comprehensive emergency care program to achieve this aim in Liberia.

Clinical outcomes associated with educational interventions are infrequently reported in existing literature. A recent study in Uganda demonstrated that three-day patient mortality during the implementation of a two-year emergency care training program for nurses in a rural emergency facility was 2.0%; however, the study did not assess mortality before program implementation for comparison [38]. A significant reduction in mortality was observed in an emergency facility in Ghana after the introduction of a unit separation model with specialty-based emergency care, but this intervention was a systems-based change as opposed to an educational intervention [39]. This is the first study to report on clinical outcomes related to the implementation of an emergency care program based on components of the WHO Basic Emergency Care toolkit. A non-significant decrease in mortality was observed, suggesting that programming did not negatively impact patient outcomes and may have improved the quality of care. The program only addressed gaps in education that were identified during the programmatic planning with stakeholders, whereas the causes of patient mortality are multifactorial and include facility infrastructure and resource availability. Redemption Hospital often lacks electricity, running water, and clean, sterile environments. Patients and their families are responsible for supplying their own food and many of their medications, and often cannot meet these needs. All these factors contribute to overall patient mortality and affect the delivery of care, likely explaining why this intervention alone did not decrease mortality. Despite this, the introduction of this program did not interrupt care so as to negatively affect patient outcomes.

This research also adds to the existing knowledge base, because emergency facility mortality in Liberia has not been previously reported. Mortality in emergency facilities in other Sub-Saharan LMICs is estimated to be 3.4% [40]. In other healthcare settings in Liberia, a wide mortality range has been described, from 23.3% at a referral hospital in Monrovia shortly after the end of the civil conflicts, to 7.4% surgical ward mortality at JFKMC, the national medical center of Liberia [41]. The high overall mortality burden illuminates a critical need for continued educational partnerships and material resources to support growth in emergency care capabilities in Liberia.

## Limitations

This study was implemented in a single center in Sub-Saharan Africa and the program was adapted to the specific needs of Redemption Hospital; therefore, the exact contents and outcomes of the program may not be generalizable to other LMIC emergency facilities. Additionally, the course relied on partnerships with visiting EM physicians, which may not be a sustainable model for longer term programming. A train the trainer model was intended, but unfortunately was not feasible during this pilot study due to unit leadership changes. As a pilot project we were limited in our scope. Future iterations must include a train the trainer model. Additionally the advent of COVID-19 made measures of sustainability and follow-up challenging. Despite these limitations, the general framework of this program is applicable to a wide variety of low-resource settings and is an important example of how basic concepts in emergency care can be implemented as part of a comprehensive curriculum using IS concepts.

Despite efforts to standardize data collection methods, inconsistent and missing records limited the quality of patient outcome data collected and may have introduced bias that affect the validity of the patient data. For example, not all patient charts were reported in the ledger introducing sampling bias. Several months of pre-course ledger data was missing and not included in analysis. Additionally, in this preliminary analysis possible confounders such as demographics, initial vitals, and chief complaint were not adjusted for due to the limitations of the existing data.

We also recognize that there is a significant risk of Hawthorne effect as the presence of having visiting faculty in the EU may have substantially led to behavior change on behalf of the participants. This effect, however, was intentional and served as a key driver of modeling behavior change. Additionally, while no substantial changes in infrastructure or personnel occurred over the study period, we also did not specifically collect and are not thought to have impacted the data in a meaningful way. Other factors may have limited the validity of the outcome measures for participant knowledge. Attendance at on-shift lectures was below 100% due to various reasons. Despite lectures being repeated several times and given at various times throughout the day to accommodate staff availability and schedules, staff were still not able to attend lectures due to low staffing in the EU and IU and importance of not disrupting patient care. In creating the program we understood that this was a potential limitation of the implementation plan and attempted to mitigate this through supplying staff with BEC workbooks and on-line AFEM lectures so they had access to all the material to review on their own. Additionally, not every visiting physician was as diligent at taking attendance during lectures and unfortunately, sign-in sheets were sometimes not utilized. Lastly, there was a component of burn-out among frontline healthcare workers as the program proceeded and they chose not to attend lectures despite being available. As participation in this program was voluntary, visiting professors tried to motivate such staff through encouragement and making the lectures engaging; however, these tactics did not always lead to participation.

Quantitative self-assessment of comfort performing emergency care tasks was used to assess programming instead of skill-based scenarios, which may have introduced bias and limited validity of the data. Skill based testing is a more objective measure, but unfortunately was not feasible given time and personnel restraints. The post-course qualitative survey was written in such a way that some of the questions could be construed as being leading to participants. This was not done intentionally and the survey questions followed a format previously used to assess self-reported knowledge and skills of healthcare workers in Liberia. Additionally, this paper does not report on overall costs related to this program and costing analysis has not been reported for *BEC*. Therefore, we cannot comment on the cost-benefit analysis of this program as compared to *BEC* as a standalone program.

Lastly, many frontline healthcare workers expressed that they lacked resources to change their practice in the EU and IU at Redemption Hospital. Although we did not report or collect data on physical resources, qualitative participant feedback suggests that investment in infrastructure and medical equipment may be needed to see additional benefits of emergency care training at Redemption Hospital.

## Conclusion

Implementation of a comprehensive six-month emergency care program specific to the local needs of a single public hospital in Liberia, using open-source AFEM and WHO tools, was feasible using an IS framework to ensure appropriate translation of evidence-based practices into the clinical environment. Using an ISLR model helped to identify key stakeholders and the RE-AIM framework grounded assessment of the program. The program was sustainable and scalable because smaller elements were taught on a repeating basis with continual assessment and improvement. Using IS frameworks, EM faculty helped evaluate and improve programming after every teaching block. Imperative next steps include making the program sustainable by training local stakeholders who can continue the iterative improvement cycle in a train the trainer model.

In summary, the course increased provider performance on validated knowledge-based exams, confidence in treating critically ill medical and trauma patients, and comfort with performing emergency care skills. EU/IU adult mortality decreased during the program implementation period, albeit not significantly. Due to the COVID-19 pandemic, longer term maintenance of the effects of this program could not be studied. This study builds on previous research supporting efforts to improve emergency care globally. Sharing these novel, adaptable strategies for implementing *BEC* and additional WHO tools provides lessons for facilities in resource-constrained environments wishing to strengthen emergency care delivery.

## Supporting information

S1 Appendix Redemption hospital triage form. (TIF)

S2 Appendix Post-course survey. (TIF)
