# Supplementary figures and images for: Implementation and outcomes of a comprehensive emergency care curriculum at a low-resource referral hospital in Liberia: A novel approach to application of the WHO Basic Emergency Care toolkit
Source: PLoS One. 2023 Mar 15;18(3):e0282690. doi: 10.1371/journal.pone.0282690 (PMC10016633; doi:10.1371/journal.pone.0282690)

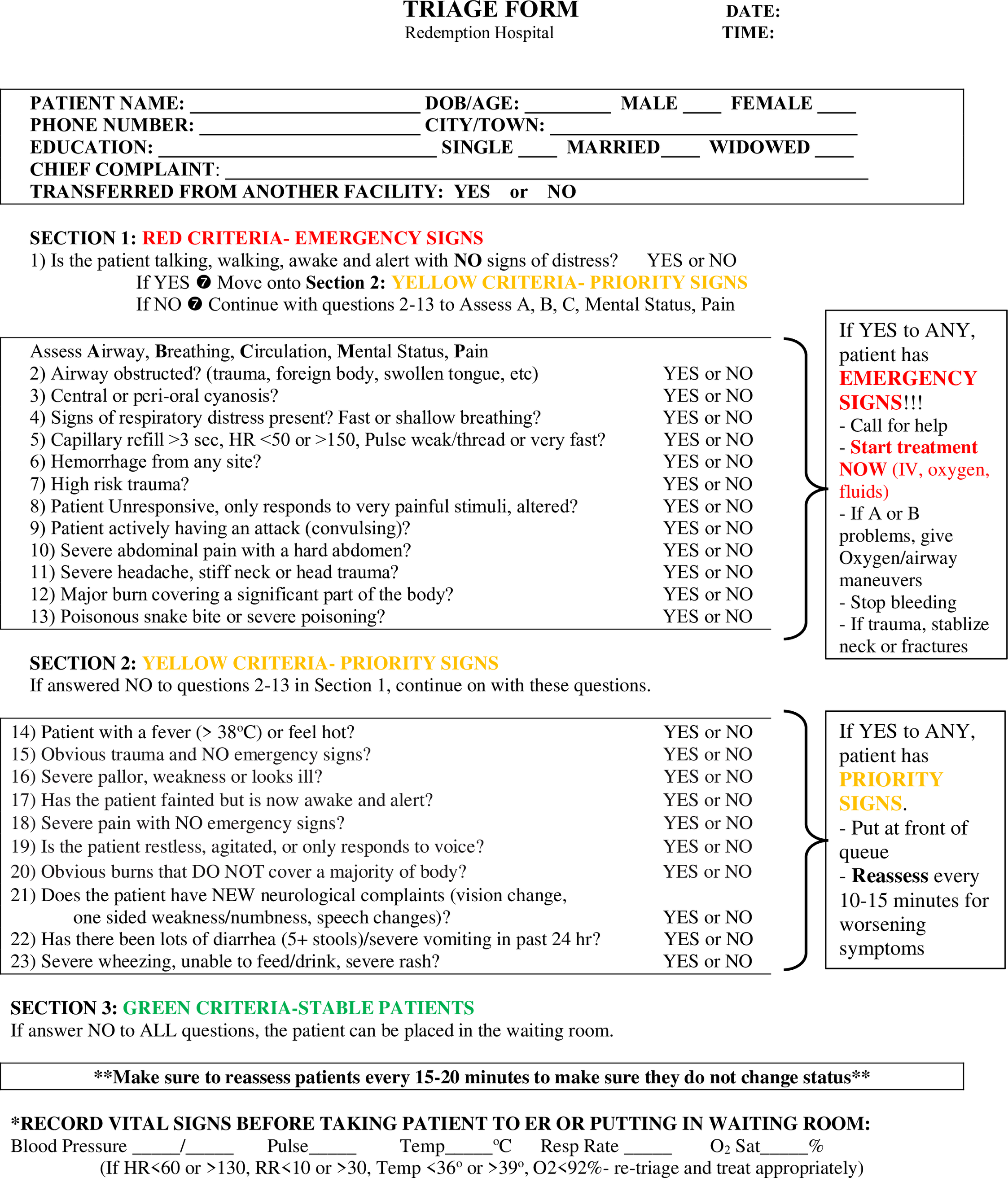

Supplement: S1 Appendix — (TIF) [file pone.0282690.s001.tif]

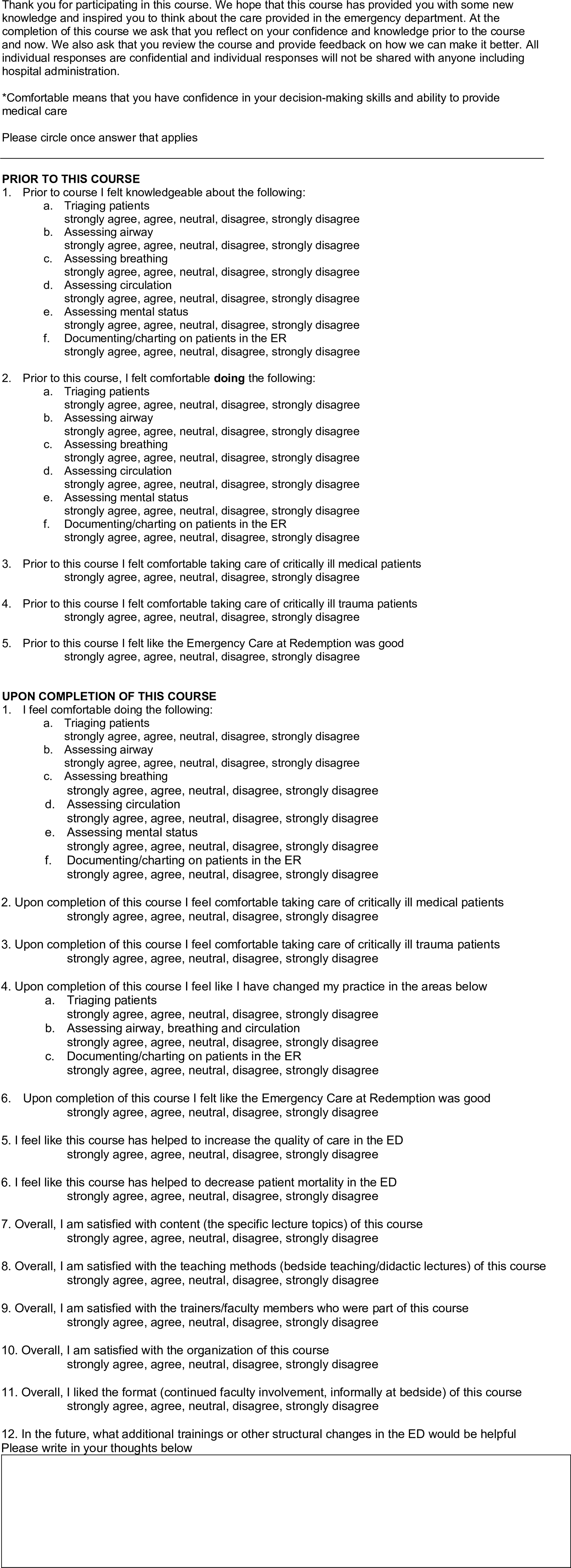

Supplement: S2 Appendix — (TIF) [file pone.0282690.s002.tif]
